# Novel genetic resources associated with sucrose and stachyose content through genome-wide association study in soybean (*Glycine max* (L.) Merr.)

**DOI:** 10.3389/fpls.2023.1294659

**Published:** 2023-11-01

**Authors:** Dongho Lee, Laura Lara, David Moseley, Tri D. Vuong, Grover Shannon, Dong Xu, Henry T. Nguyen

**Affiliations:** ^1^Fisher Delta Research, Extension, and Education Center, Division of Plant Science and Technology, University of Missouri, Portageville, MO, United States; ^2^Division of Plant Science and Technology, University of Missouri, Columbia, MO, United States; ^3^Agrícola Los Alpes, Chimaltenango, Guatemala; ^4^Dean Lee Research and Extension Center, LSU AgCenter, Alexandria, LA, United States; ^5^Department of Electrical Engineering and Computer Sciences, Christopher S. Bond Life Science Center, University of Missouri, Columbia, MO, United States

**Keywords:** genome-wide association study, RNA-Seq Atlas, soybean, soluble carbohydrate, quantitative trait loci

## Abstract

The nutritional value of soybean [*Glycine max* (L.) Merr.] for animals is influenced by soluble carbohydrates, such as sucrose and stachyose. Although sucrose is nutritionally desirable, stachyose is an antinutrient causing diarrhea and flatulence in non-ruminant animals. We conducted a genome-wide association study of 220 soybean accessions using 21,317 single nucleotide polymorphisms (SNPs) from the SoySNP50K iSelect Beadchip data to identify significant SNPs associated with sucrose and stachyose content. Seven significant SNPs were identified for sucrose content across chromosomes (Chrs.) 2, 8, 12, 17, and 20, while thirteen significant SNPs were identified for stachyose content across Chrs. 2, 5, 8, 9, 10, 13, 14, and 15. Among those significant SNPs, three sucrose-related SNPs on Chrs. 8 and 17 were novel, while twelve stachyose-related SNPs on Chrs. 2, 5, 8, 9, 10, 13, 14, and 15 were novel. Based on Phytozome, STRING, and GO annotation, 17 and 24 candidate genes for sucrose and stachyose content, respectively, were highly associated with the carbohydrate metabolic pathway. Among these, the publicly available RNA-seq Atlas database highlighted four candidate genes associated with sucrose (*Glyma.08g361200* and *Glyma.17g258100*) and stachyose (*Glyma.05g025300* and *Glyma.13g077900*) content, which had higher gene expression levels in developing seed and multiple parts of the soybean plant. The results of this study will extend knowledge of the molecular mechanism and genetic basis underlying sucrose and stachyose content in soybean seed. Furthermore, the novel candidate genes and SNPs can be valuable genetic resources that soybean breeders may utilize to modify carbohydrate profiles for animal and human usage.

## Introduction

Soybean [*Glycine max* (L.) Merr.] is one of the most economically valuable crops, providing high protein meal and vegetable oil for human and animal diets worldwide. Annually, almost 76% of global soybean production is used to feed livestock for the meat and dairy industries (Ritchie and Roser, 2021). In comparison, 20% and 4% of production are directly used for the human diet and biofuel industry, respectively (Ritchie and Roser, 2021). In 2021, the U.S., the world’s second soybean producer, produced almost 46 million metric tons of soybean meal, which was fed to poultry (61.2%), swine (18.0%), dairy (13.4%), beef (5.1%), and others (2.3%) (American Soybean Association, 2021). Recently, soy-based products have garnered more attention from the global vegan population as a high-value protein substitute for animal meats (Qin et al., 2022). Also, the number of companion pet owners who prefer plant-based pet foods, such as soybean, over animal-based products has been growing due to animal welfare, ethical, and moral concerns (Yoosefzadeh-Najafabadi et al., 2022). In the U.S., the pet industry is an important market that has been booming for decades, of which approximately 66% of U.S. households own a pet and spend almost 58.1 billion dollars on pet food and treats annually (APPA, 2023).

Soybean seed typically consists of 40% protein, 20% oil, and 15% soluble carbohydrates on a dry weight basis (Hsu et al., 1973). While soybean meal is high in crude protein content with well-balanced amino acids for animal feeds, some antinutrients in soluble carbohydrates significantly reduce feed efficiency for non-ruminant animals, including poultry, swine, dogs, cats, and humans (Jo et al., 2018; Cunicelli et al., 2019; Jo et al., 2019). Sucrose is the only soluble carbohydrate nutritionally beneficial to produce metabolizable energy. In contrast, raffinose and stachyose make up the raffinose family of oligosaccharides (RFOs), known as antinutrients causing diarrhea and flatulence in non-ruminant animals (Liu, 1997; Guillon and Champ, 2002; Karr-Lilienthal et al., 2005). Non-ruminant animals lack α-galactosidase in their digestive systems, in which undigested RFOs pass through the upper intestine and are fermented by anaerobic microbes in the lower intestine. This produces methane, hydrogen, and carbon dioxide that cause gastric discomfort and a significant loss of energy efficiency from the soybean meal (Coon et al., 1990; Le et al., 2020; Salari et al., 2021). Since the largest soybean meal consumers are non-ruminant animals, developing new soybean cultivars with high sucrose and low RFOs is crucial to improve digestibility and feed efficiency. In addition, increased sucrose content in soybean seeds is also essential to improve the sweet flavor of soy-based products, such as tofu, edamame, and soymilk (Rosset et al., 2012; Sui et al., 2020; Wang et al., 2023).

Compared to other seed compositional traits in soybeans, such as oil and protein, a relatively smaller number of quantitative trait loci (QTL) for soluble carbohydrates have been identified and reported through genetic linkage analysis. Historically, Maughan et al. (2000) first reported 17 QTL related to sucrose across chromosomes (Chrs.) 5, 7, 8, 13, 15, 19, and 20 using 149 F_2_ individuals from an interspecific cross between *G. max* and *G. soja*. Other studies in South Korea reported four sucrose- and two oligosaccharides-related QTL on Chrs. 2, 11, and 19 and Chrs. 2 and 19, respectively. Two common QTL on Chrs. 2 and 19 were found for both traits in the RIL population (Kim et al., 2005). A year later, Kim et al. (2006) identified two sucrose- and four oligosaccharides-related QTL on Chrs. 12 and 16 and Chrs. 6, 12, 16, and 19, respectively. Two QTL on Chrs. 12 and 16 were identified for both traits. Skoneczka et al. (2009) analyzed two F_2_ populations and identified a major QTL on Chr. 6 for sucrose and stachyose, which explained 76% and 88% of the phenotypic variations, respectively. Saghai Maroof and Buss (2011) found a major QTL related to both sucrose and stachyose on Chr. 11. Using the F_2_ population derived from the same high sucrose soybean line used by Saghai Maroof and Buss (2011); Wang et al. (2014) identified three sucrose-related QTL on Chrs. 7, 11, and 20 and two stachyose-related QTL on 11 and 12. The QTL on Chr. 11 was in the same genetic region reported by Saghai Maroof and Buss (2011). Zeng et al. (2014) reported three novel QTL on Chrs. 5, 9, and 16, explaining 46%, 10%, and 8% of sucrose variation, respectively. Akond et al. (2015) found three sucrose- and four stachyose-associated QTL on Chrs. 3, 9, and 15 and 1, 6, 12, and 14, respectively. Patil et al. (2018) identified three QTL on Chrs. 6, 16, and 20, and a major QTL on Chr. 8 for sucrose using an interspecific population derived from a cross between *G. max* and *G. soja* accessions.

Genome-wide association study (GWAS) is a valid alternative to genetic linkage analysis to understand the genetic basis of quantitative traits by examining a significant association between genetic markers and a trait of interest. To date, GWAS has been successfully applied in soybean research to discover and characterize key traits, such as seed protein and oil (Hwang et al., 2014; Li et al., 2019; Zhang et al., 2021), amino acids (Lee et al., 2019; Yuan et al., 2021), fatty acids (Liu et al., 2020; Sung et al., 2021), disease resistance (Vuong et al., 2015; Ravelombola et al., 2020; Vieira et al., 2022; McDonald et al., 2023), abiotic stress tolerance (Kaler et al., 2017; Wu et al., 2019; Saleem et al., 2022), agronomic traits (Ayalew et al., 2022; Cao et al., 2022; Yang et al., 2022), and root system (Seck et al., 2020; Rathore et al., 2022; Kim et al., 2023). However, only a few studies have implemented GWAS for soluble carbohydrates in soybean seeds (Lu et al., 2022; Xu et al., 2022).

In this study, a diverse panel of 220 soybean accessions and 21,317 polymorphic single nucleotide polymorphisms (SNPs) were used to conduct GWAS to identify significant marker-trait associations for sucrose and stachyose through a mixed linear model (MLM). Among the three main soluble carbohydrates, raffinose was excluded from this study due to the little phenotypic variation and non-Gaussian distribution in the accession panel. The gene function, protein interaction, biochemical pathway, and gene expression of potential candidate genes associated with sucrose and stachyose content were further studied.

## Materials and methods

### Accession panel selection and field experimental design

A diverse panel of 220 soybean plant introductions (PIs) was selected based on the 100-seed weight (> 23 g) and relevant maturity groups (MGs) from the USDA-ARS Soybean Germplasm Collection (https://www.ars-grin.gov/) (**Supplementary Table 1**). The panel included four MGs, III, IV, V, and VI, that originated from six countries (China, Japan, North Korea, South Korea, Taiwan, and the United States). The panel was grown at the Arkansas Agricultural Research and Extension Center (36.06 °N 94.16 °W) in Fayetteville, AR, and the Rice Research and Extension Center (34.47 °N 91.41 °W) in Stuttgart, AR, in 2014 (FAY_14 and STU_14, respectively) and 2015 (FAY_15 and STU_15, respectively). Each accession was planted in single 3-m rows spaced 75 cm apart in a randomized complete block design (RCBD) with two replications. In 2020 and 2021, the accessions were grown at the Fisher Delta Research, Extension, and Education Center (FDREEC) (36.42 °N 89.70 °W) in Portageville, MO (POR_20 and POR_21) and the Bradford Research and Extension Center (BREC) (38.89 °N 92.19 °W) in Columbia, MO (COL_21). Ten seeds of each accession were planted in 75 cm wide rows in hill plots spaced 30 cm apart at the FDREEC and 60 cm apart at the BREC. The experiments at FDREEC and BREC were planted in a RCBD design with two replications.

### Soluble carbohydrate phenotyping

Soybean seeds of each accession in each replication were harvested at maturity. Ten seeds were sampled per plot to quantify soluble carbohydrates in the Soybean Genetics & Genomics Laboratory under the supervision of Dr. Henry Nguyen at the University of Missouri, Columbia. The content of soluble carbohydrates was measured using the established High-Performance Liquid Chromatography (HPLC) protocol described by Valliyodan et al. (2015). Briefly, around 1 g of soybean seeds was ground using Thomas Wiley Mini-Mill (Arthur Thomas Co., Chadds Ford, PA, USA) fitted with a 20-mesh screen. The soybean powder was then lyophilized for 48 hours using a Labconco freeze-dry system (Labconco, Kansas City, MO, USA). Precisely, 90.25 (± 0.15) mg of dried soybean powder was mixed with 900 μL HPLC-grade water in a 2 mL centrifuge tube. Each tube was incubated at 55°C, agitated at 200 rpm for an hour, and then vortexed for 30 seconds. After 20 minutes under room temperature, 900 μL HPLC- grade acetonitrile was added to each tube. Next, the suspension was centrifuged for 30 minutes at a 14.0 × 1000 min^−1^ × g speed. The supernatant was diluted five times with 65% HPLC-grade acetonitrile to prepare the final sample. The final samples were loaded on the Agilent HPLC-ELSD (Evaporative Light Scattering Detection) 120 series (Agilent, Santa Clara, CA, USA), equipped with the Prevail Carbohydrate ES columns (5 μm 250 × 4.6 mm) and guard columns (7.5 × 4.6 mm) (Grace Davison Discovery Sciences, Deerfield, IL, USA). Standard mixtures were prepared in HPLC-grade water with 50, 100, 300, 500, and 1000 μg/mL concentrations to create calibration curves.

### Statistical analysis

Analysis of variance (ANOVA) was conducted using the GLM procedure of SAS software version 9.4 with ‘*Genotype’* within ‘*Maturity group*’ as fixed effects and ‘*Environment*’, ‘*Maturity group*’, ‘*Genotype × Environment*’, and ‘*Replication*’ as random effects. The best linear unbiased prediction (BLUP) was computed using the *lmer* function in R software to minimize the effects of environmental variation and used as an additional environment in GWAS analysis. Pearson’s correlation coefficients between sucrose and stachyose were calculated using the *chart.Correlation* function in R software across environments and within each environment. The significant difference in mean values across seven environments between favorable and unfavorable alleles was determined using the PROC ANOVA function in the SAS software.

### Genotype data processing

The SoySNP50K iSelect Beadchip data for the 220 PI lines were obtained from Soybase (https://www.soybase.org/). The SNPs with a minor allele frequency (MAF) less than 0.05 were removed using GAPIT (Lipka et al., 2012). A total of 21,317 SNPs were used for GWAS in this study. The number of filtered SNPs mapped across 20 soybean chromosomes ranged from 693 on Chr. 20 to 1,677 on Chr. 18, with an average of 1,066 SNPs (**Supplementary Figure 1**).

### Population structure analysis and linkage disequilibrium estimation

Population structure was analyzed using the STRUCTURE software version 2.3.4 (Pritchard et al., 2000). The hypothetical number of subpopulations (K) from 2 to 9 was set with five independent iterations. For each run, the burn-in iteration and Markov Chain Monte Carlo replication were set at 10,000 and 25,000, respectively. Principal component analysis (PCA) was performed using TASSEL software version 5.0 (Bradbury et al., 2007). The Linkage disequilibrium (LD) block was calculated by computing correlation coefficients (*r^2^*) for all pairwise marker comparisons and visualized using Haploview software to identify potential candidate genes (Barrett et al., 2005). The kinship matrix was also generated by centered-IBS methods using TASSEL software.

### Genome-wide association studies

Principal components (PCs) and a kinship matrix were incorporated in MLM as covariates to correct for population structure and cryptic relatedness. The MG was additionally used as a categorical covariate since the MG effects were significant on sucrose and stachyose based on ANOVA. The significant threshold of SNP-trait association at a *P*-value = 1.0 x 10^-3^ was suggested to identify consistent and significant SNPs across all environments. The GWAS was conducted for each environment and BLUP. Significant SNPs were determined when the SNP was identified in three or more environments. If multiple significant SNPs were detected within the same LD block, the most consistent SNP with the highest -log_10_(P) value was selected.

### The gene function, protein interaction, and biochemical pathway of candidate genes

All potential candidate genes within the LD block of each significant SNP were obtained using *Glycine max* cv. Williams 82 reference-genome gene models version 2.0 in Soybase. Also, the predicted amino acid sequences of the potential candidate genes were obtained from Soybase and used to study the protein interaction network using the STRING database (https://string-db.org/) (Szklarczyk et al., 2019). Relevant candidate genes were selected based on the metabolic studies of carbohydrates. The most adjacent candidate gene was selected if no relevant candidate gene was found within the LD block. Gene ontology (GO) annotation was obtained to confirm the biological processes, cellular components, and molecular functions of the relevant candidate genes using Database for Annotation, Visualization and Integrated Discovery (DAVID) bioinformatics resources (http://david.ncifcrf.gov/) (Sherman et al., 2021).

### Tissue-specific gene expression analysis

The RNA-Seq Atlas data, publicly available on Soybase (http://soybase.org/soyseq/), were used to compare the gene expression levels between the relevant candidate genes in 14 different soybean plant tissues, including young leaf, flower, 1-cm pod, pod shell 10 days after flowering (DAF), pod shell 14 DAF, seed 10 DAF, seed 14 DAF, seed 21 DAF, seed 25 DAF, seed 28 DAF, seed 35 DAF, seed 42 DAF, root, and nodule (Severin et al., 2010). The raw gene expression counts were normalized using a Reads Per Kilobase of transcript per Million mapped reads (RPKM) method. Highly expressed candidate genes were determined based on at least two gene expression levels of more than 10 RPKM in developing seed tissues (https://www.ebi.ac.uk/). Another large set of RNA-Seq databases of gene-level transcript abundances (https://soyatlas.venanciogroup.uenf.br/) was used for additional exploration in the differential gene expression across 19 parts of the soybean plant, including cotyledon, embryo, endosperm, epicotyl, flower, hypocotyl, leaf, nodule, petiole, pod, radicle, root, seed, seed coat, seedling, shoot, suspensor, unknown, and whole plant (Almeida-Silva et al., 2023). The raw gene expression counts were normalized using a Transcripts Per Million (TPM) method. Highly expressed candidate genes were determined based on the total gene expression level of more than 1000 TPM across all parts (https://www.ebi.ac.uk/).

## Results

### Evaluations of phenotypic data

The panel of 220 soybean accessions was tested for sucrose and stachyose content in seven environments. A summary of the phenotypic values of each environment is shown in **Table 1**. The highest mean value for sucrose was 7.3% in POR_20, with a range of 4.5% - 9.5%, while the lowest was 5.8% in STU_15, with a range of 4.1% - 8.4%. The coefficients of variation (CV) ranged from 11.0% (POR_20) to 14.4% (STU_14), and Shapiro-Wilk (w) values ranged from 0.974 (STU_15) to 0.995 (FAY_15). The highest mean value for stachyose was 4.5% in STU_14, with a range of 2.2% - 6.3%, while the lowest mean was 3.5% in FAY_15, with a range of 1.0% - 5.6%. The CV ranged from 16.2% (POR_21) to 25.5% (FAY_15), and Shapiro-Wilk (w) values ranged from 0.940 (STU_15) to 0.991 (FAY_15). Pearson’s correlations among environments for sucrose content varied from 0.26 (between STU_14 and POR_21) to 0.77 (between FAY_14 and STU_14) (**Supplementary Figure 2**). Pearson’s correlations among environments for stachyose content varied from 0.64 (between STU_14 and POR_21 and between STU_15 and COL_21) to 0.93 (between FAY_15 and POR_20). Also, Pearson’s correlation between sucrose and stachyose content was estimated to be significantly negative in three environments (FAY_14, FAY_15, and STU_15) and significantly positive in one environment (STU_14) (**Supplementary Table 2**). The ANOVA showed significant effects for ‘*Genotype*’ within maturity groups, ‘*Environment*’, ‘*Maturity group*’, and ‘*Genotype X Environment*’ for sucrose and stachyose content but no significant effect for ‘*Replication*’ (**Supplementary Table 3**).

**Table 1:** Summary of sucrose and stachyose content in seven environments.

Trait	Env^a^	Range (%)	Mean (SE)^b^	CV^c^	Shapiro-Wilk (w)	Skewness	Kurtosis
Sucrose	FAY_14	4.5 - 9.1	6.9 (0.057)b	12.2	0.992	-0.01	3.21
	STU_14	3.9 - 8.9	6.4 (0.062)d	14.4	0.990	0.09	2.84
	FAY_15	4.5 - 8.8	6.7 (0.053)c	11.7	0.995	-0.18	2.97
	STU_15	4.1 - 8.4	5.8 (0.049)f	12.4	0.974	0.56	4.03
	POR_20	4.5 - 9.5	7.3 (0.054)a	11.0	0.989	-0.28	3.45
	COL_21	4.1 - 9.0	6.1 (0.056)e	13.5	0.987	0.32	3.18
	POR_21	4.1 - 9.3	6.4 (0.062)d	14.2	0.991	-0.08	2.79
Stachyose	FAY_14	1.1 - 5.6	3.9 (0.061)b	23.2	0.948	-0.85	3.69
	STU_14	2.2 - 6.3	4.5 (0.052)a	16.9	0.984	-0.24	3.46
	FAY_15	1.0 - 5.6	3.5 (0.059)c	25.5	0.991	-0.13	2.88
	STU_15	1.3 - 5.5	3.9 (0.053)b	20.2	0.940	-0.92	4.19
	POR_20	1.4 - 5.4	3.6 (0.051)c	20.9	0.987	-0.27	3.19
	COL_21	2.0 - 5.6	4.1 (0.045)b	16.3	0.984	-0.34	3.21
	POR_21	2.0 - 5.5	4.0 (0.044)b	16.2	0.958	-0.55	3.53

^a^Environments. FAY_14 (Fayetteville in 2014), STU_14 (Stuttgart in 2014), FAY_15 (Fayetteville in 2015), STU_15 (Stuttgart in 2015), POR_20 (Portageville in 2020), COL_21 (Columbia in 2021), POR_21 (Portageville in 2021); ^b^Different alphabet letters within the trait indicate mean values are significantly different at p < 0.05. SE indicates a standard error; ^c^Coefficients of variation.

### Population structure

**Figure 1:**
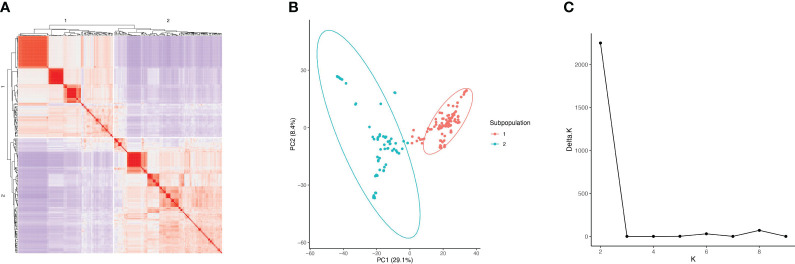
Kinship and population structure analysis of 220 soybean accessions. **(A)** Kinship matrix visualized in the heat map. **(B)** PCA plot of the 220 soybean accessions. **(C)** The optimal number of subpopulations (K) in the accession panel.

The cryptic relatedness among 220 soybean accessions, estimated by kinship matrix and visualized in the heat map, indicated two distinct subpopulations (1 and 2) (**Figure 1A**). The PCA plot demonstrated that the two components accounted for 29.1% (PC1) and 8.4% (PC2) of genetic variation, which differentiate the two subpopulations (**Figure 1B**). The first subpopulation had 116 accessions with origins from China (4), Japan (79), North Korea (1), South Korea (22), Taiwan (6), and the United States within MG III (30), IV (43), V (21), and VI (22). The second subpopulation had 104 accessions with origins from China (2), Japan (2), North Korea (5), and South Korea (95) within MG III (1), IV (100), V (2), and VI (1). STRUCTURE analysis also suggested that the optimal number of subpopulations (K) was two among all genotypes (**Figure 1C**).

### Association study for sucrose and stachyose content

**Figure 2:**
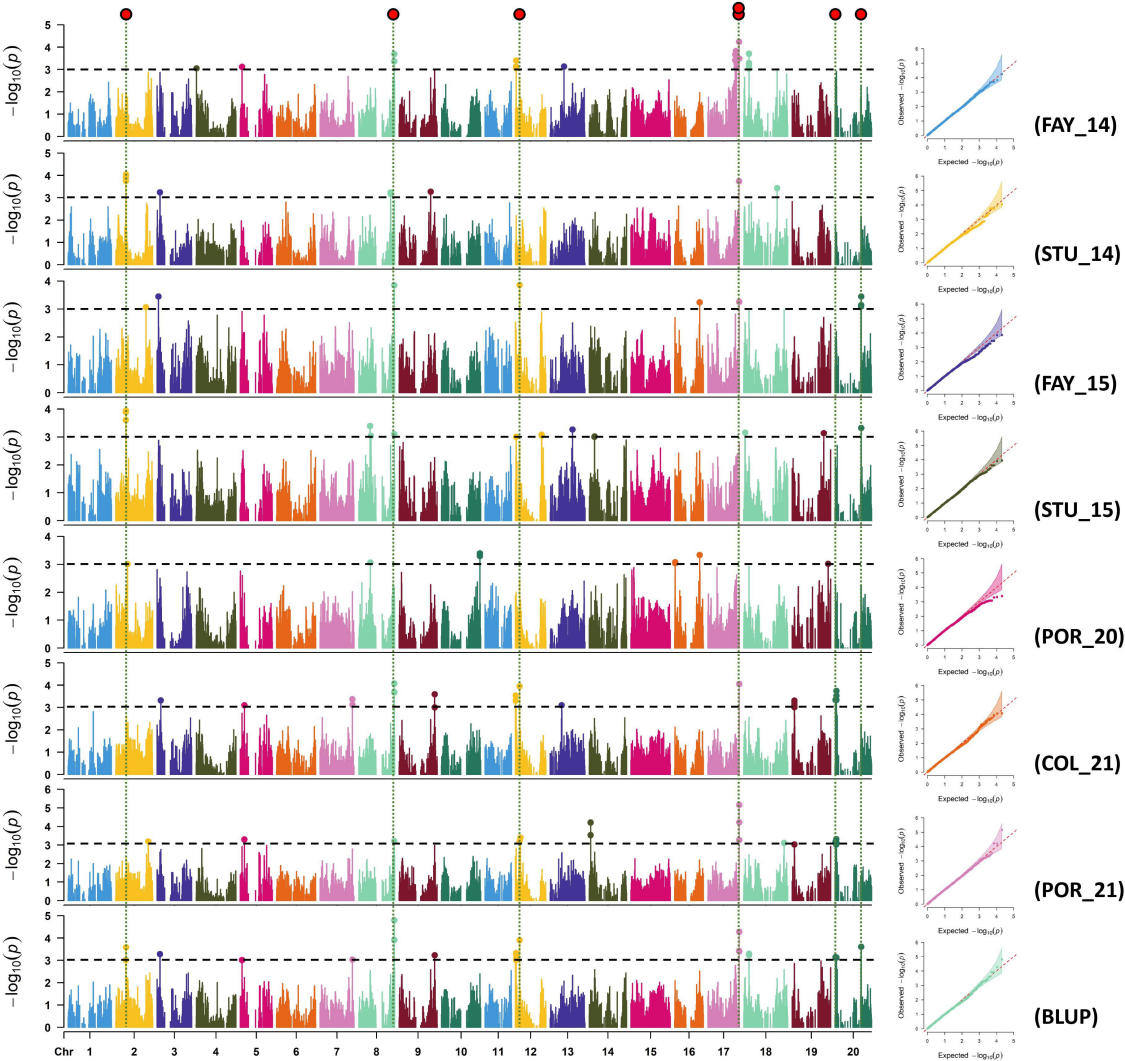
Manhattan and quantile-quantile plots of sucrose for FAY_14 (Fayetteville in 2014), STU_14 (Stuttgart in 2014), FAY_15 (Fayetteville in 2015), STU_15 (Stuttgart in 2015), POR_20 (Portageville in 2020), COL_21 (Columbia in 2021), POR_21 (Portageville in 2021), and BLUP (across seven environments, an additional environment). SNP markers detected in three or more environments were considered significant SNPs using GWAS threshold -log_10_(P) > 3 (red dots on the top).

**Figure 3:**
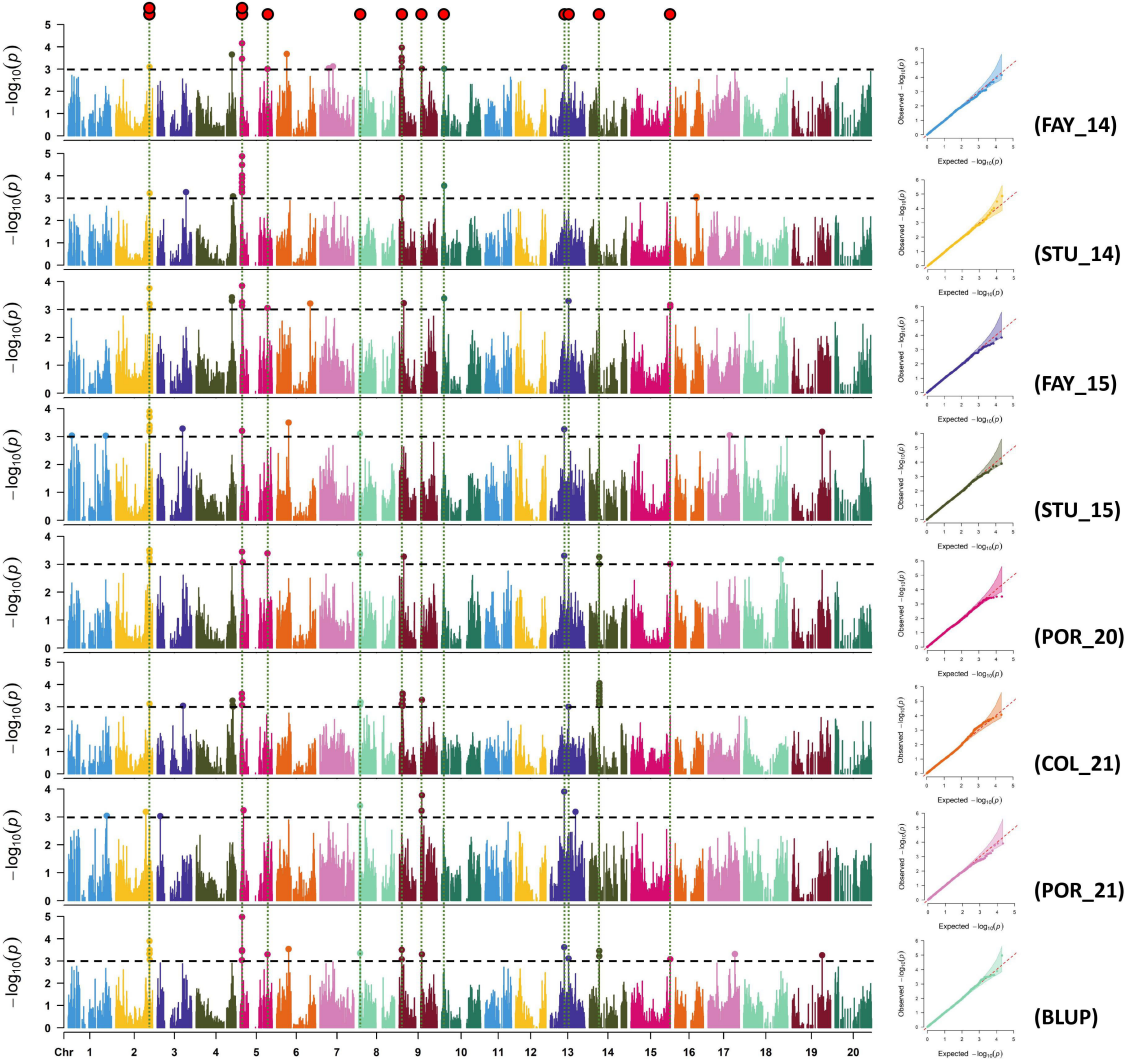
Manhattan and quantile-quantile plots of stachyose for FAY_14 (Fayetteville in 2014), STU_14 (Stuttgart in 2014), FAY_15 (Fayetteville in 2015), STU_15 (Stuttgart in 2015), POR_20 (Portageville in 2020), COL_21 (Columbia in 2021), POR_21 (Portageville in 2021), and BLUP (across seven environments, an additional environment). SNP markers detected in three or more environments were considered significant SNPs using GWAS threshold -log_10_(P) > 3 (red dots on the top).

Manhattan and quantile-quantile (Q-Q) plots for each environment for sucrose and stachyose content are shown in **Figures 2**, **3**, respectively. Across all environments, a total of 88 and 89 SNPs were associated with sucrose and stachyose content, respectively (**Supplementary Table 4**).

Seven out of 88 SNPs, identified in three or more environments for sucrose content, were considered significant SNPs. The seven significant SNPs were located on Chrs. 2 (ss715581183), 8 (ss715602502), 12 (ss715613179), 17 (ss715627820 and ss715627853), and 20 (ss715636857 and ss715637428) (**Figure 2**; **Table 2**). The SNP ss715602502 located at 47,286,262 bp on Chr. 8, identified in five environments (FAY_14, FAY_15, STU_15, COL_21, and BLUP), was the most consistent SNP for sucrose content. The SNPs ss715613179 located at 5,486,355 bp on Chr. 12 were identified in four environments: FAY_15, COL_21, POR_21, and BLUP. The SNP ss715627853 located at 41,440,620 bp on Chr. 17 were also identified in four environments: FAY_14, COL_21, POR_21, and BLUP (**Table 2**; **Supplementary Table 4**). The highest -log_10_ (P) value (5.2) was found on the SNP ss715627853 on Chr. 17. The MAF of the significant SNPs ranged from 0.05 (ss715627820) to 0.39 (ss715636857) (**Table 2**).

**Table 2:** Significant SNPs associated with soybean sucrose and stachyose content identified in three or more environments using a mixed linear model.

Trait	Significant SNP^a^	Chr^b^	Position (bp)	# Env^c^	Allele^d^		-log_10_ (P)	MAF^e^
					Favorable	Unfavorable		
Sucrose	ss715581183	2	13,523,639	3	G	T	3.6 - 4.0	0.37
	ss715602502	8	47,286,262	5	G	T	3.1 - 4.8	0.18
	ss715613179	12	5,486,355	4	C	T	3.2 - 4.0	0.33
	ss715627820	17	41,098,767	3	A	G	3.4 - 4.2	0.05
	ss715627853	17	41,440,620	4	T	C	3.5 - 5.2	0.13
	ss715636857	20	1,907,881	3	G	A	3.1 - 3.7	0.39
	ss715637428	20	34,286,637	3	T	C	3.3 - 3.6	0.07
Stachyose	ss715583079	2	44,214,908	3	G	A	3.3 - 3.7	0.12
	ss715583119	2	44,448,179	6	T	G	3.1 - 3.9	0.11
	ss715592340	5	2,207,089	3	G	A	3.0 - 3.4	0.20
	ss715592442	5	2,369,980	7	C	A	3.2 - 5.0	0.18
	ss715591198	5	35,773,064	4	T	G	3.0 - 3.4	0.16
	ss715601133	8	2,382,910	4	T	C	3.1 - 3.4	0.21
	ss715603880	9	3,771,212	3	C	T	3.0 - 4.0	0.32
	ss715639178	9	30,134,957	4	A	G	3.0 - 3.8	0.47
	ss715606330	10	3,541,231	3	C	T	3.0 - 3.6	0.08
	ss715615716	13	18,379,941	5	C	T	3.1 - 3.9	0.09
	ss715614101	13	24,090,619	3	G	A	3.0 - 3.3	0.14
	ss715617675	14	13,187,218	3	C	T	3.3 - 3.9	0.29
	ss715622806	15	51,630,810	3	G	A	3.0 - 3.2	0.31

^a^Single-nucleotide polymorphism; ^b^Chromosome; ^c^The number of environments where significant SNP was identified, a total number of environments were eight; ^d^The allele conferring higher sucrose content was designated a favorable allele, while the allele conferring lower stachyose content was designated a favorable allele; ^e^Minor allele frequency.

Thirteen out of 89 SNPs, identified in three or more environments for stachyose content, were considered significant SNPs. The thirteen significant SNPs were located on Chrs. 2 (ss715583079 and ss715583119), 5 (ss715592340, ss715592442, and ss715591198), 8 (ss715601133), 9 (ss715603880 and ss715639178), 10 (ss715606330), 13 (ss715614101 and ss715615716), 14 (ss715617675), and 15 (ss715622806) (**Figure 3**; **Table 2**). The SNP ss715592442 located at 2,369,980 bp on Chr. 5, identified in seven environments (FAY_14, STU_14, FAY_15, STU_15, POR_20, COL_21, and BLUP), was the most consistent SNP for stachyose content. The SNP ss715583119 located at 44,448,179 bp on Chr. 2 for stachyose content was identified in six environments: FAY_14, FAY_15, STU_15, POR_20, COL_21, and BLUP (**Table 2**; **Supplementary Table 4**). The highest -log_10_ (P) value (5.0) was found on the SNP ss715592442 on Chr. 5. The MAF of the significant SNPs ranged from 0.08 (ss715606330) to 0.47 (ss715639178) (**Table 2**).

### Allelic effect of significant SNPs for sucrose and stachyose content

**Figure 4:**
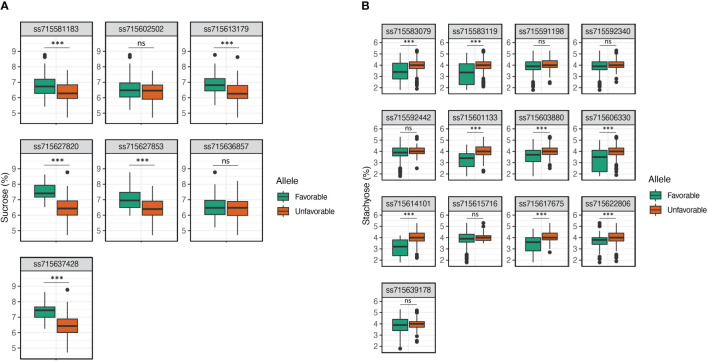
Allelic effect of significant SNPs for sucrose **(A)** and stachyose **(B)** content using mean values of favorable and unfavorable alleles across seven environments. The allele conferring higher sucrose content was designated a favorable allele, while the allele conferring lower stachyose content was designated a favorable allele. ns, no significant; *, significant level *p* < 0.05; **, significant level *p* < 0.01; and ***, significant level *p* < 0.001.

The allelic effects of significant SNPs for sucrose and stachyose content were tested using mean values of favorable and unfavorable alleles across seven environments (**Figure 4**). An allele conferring higher sucrose content was designated a favorable allele. In contrast, an allele conferring lower stachyose content was designated a favorable allele. The favorable alleles of five SNPs, ss715581183 (Chr. 2), ss715613179 (Chr. 12), ss715627820 (Chr. 17), ss715627853 (Chr. 17), and ss715637428 (Chr. 20), were related to significantly higher sucrose content than unfavorable alleles (*p*-value < 0.001) (**Figure 4A**). However, two SNPs, ss715602502 (Chr. 8) and ss715636857 (Chr. 20), showed no significant difference between favorable and unfavorable alleles for sucrose content. The favorable alleles of eight SNPs, ss715583079 (Chr. 2), ss715583119 (Chr. 2), ss715601133 (Chr. 8), ss715603880 (Chr. 9), ss715606330 (Chr. 10), ss715614101 (Chr. 13), ss715617675 (Chr. 14), and ss715622806 (Chr. 15), were related to significantly lower stachyose content than unfavorable alleles (*p*-value < 0.001) (**Figure 4B**). However, the five SNPs, ss715592340 (Chr. 5), ss715592442 (Chr. 5), ss715591198 (Chr. 5), ss715639178 (Chr. 9), and ss715615716 (Chr. 13), showed no significant difference between favorable and unfavorable alleles for stachyose content. SNPs having no significant allelic effects, however, still showed favorable phenotypic trends across environments (**Supplementary Figure 3**). To evaluate the pyramiding effects of favorable alleles of significant SNPs, the variations of sucrose and stachyose content across seven environments were compared in the different numbers of favorable alleles (**Table 3**; **Supplementary Table 5**). The results showed that sucrose contents ranged from 5.7% ± 0.7 (none) to 7.9% ± 0.4 (six favorable alleles), and stachyose contents ranged from 2.0% ± 0.2 (13 favorable alleles) to 5.3% ± 0.0 (one favorable allele).

**Table 3:** The phenotypic variations of sucrose and stachyose contents across seven environments in the different numbers of favorable alleles of significant SNPs.

Trait	No. of favorable alleles	No. of genotypes	Mean ± SD^a^
			(%)
Sucrose	0	10	5.7 ± 0.7
	1	50	6.5 ± 0.5
	2	72	6.3 ± 0.6
	3	45	6.5 ± 0.4
	4	32	7.0 ± 0.6
	5	8	7.7 ± 0.5
	6	3	7.9 ± 0.4
Stachyose	1	1	5.3 ± 0.0
	2	3	4.4 ± 0.8
	3	26	4.1 ± 0.5
	4	49	4.1 ± 0.4
	5	82	4.1 ± 0.6
	6	13	3.9 ± 0.4
	7	13	3.4 ± 0.8
	8	10	3.1 ± 0.5
	9	13	3.5 ± 0.8
	10	2	3.0 ± 0.3
	11	3	3.1 ± 1.3
	12	2	2.2 ± 0.2
	13	3	2.0 ± 0.2

^a^Standard deviation.

### Prediction of potential candidate genes for sucrose and stachyose content

A total of 107 and 155 genes located within the LD blocks of significant SNPs were considered potential candidate genes for sucrose and stachyose, respectively (**Supplementary Table 6**). The estimated size of LD blocks varied from 6 Kbp to 528 Kbp with an average of 186 Kbp, which is slightly longer than that of previously tested cultivated soybeans (~150 Kbp) (Lam et al., 2010) (**Supplementary Table 6**).

Out of 107 genes, 17 relevant candidate genes for sucrose content were closely associated with carbohydrate metabolism (**Table 4**). Since no candidate gene related to carbohydrate metabolism was located within the LD block of ss715637428 for sucrose content, the most adjacent candidate gene to the significant SNP, *Glyma.20g099600*, was selected. The largest number of relevant candidate genes (seven) were found in the LD block of ss715602502 on Chr. 8 related to three sugar transporter-related genes, *Glyma.08g360400*, *Glyma.08g360500*, and *Glyma.08g361200*, and four other genes, *Glyma.08g356800*, *Glyma.08g357200*, *Glyma.08g358700*, and *Glyma.08g358800*, that interact with carbohydrate metabolism-related proteins (polygalacturonase, 1D-myo-inositol 3-kinase, mannosyl-oligosaccharide α-1,3-glucosidase, and UDP-sugar pyrophosphorylase, respectively). Also, four genes, *Glyma.12g072800*, *Glyma.20g017400*, *Glyma.20g018000*, and *Glyma.20g018200*, are involved in carbohydrate metabolism, while five genes, *Glyma.02g129200*, *Glyma.17g257800*, *Glyma.17g258100*, *Glyma.17g260300*, and *Glyma.17g260400*, have functional interactions with carbohydrate metabolism-related proteins.

**Table 4:** The list of candidate genes associated with carbohydrate metabolic pathways, identified within the linkage disequilibrium block of significant SNPs.

Trait	Significant SNP^a^	Chr^b^	Position (bp)	Candidate gene^c^	Function annotation^d^	Protein interaction^e^
Sucrose	ss715581183	2	13,523,639	*Glyma.02g129200*	Predicted hydrolases of HD superfamily	Xyloglucan galactosyltransferase
	ss715602502	8	47,286,262	*Glyma.08g356800*	Pectin lyase-like superfamily protein	Polygalacturonase
				*Glyma.08g357200*	Serine-Threonine protein kinase	1D-myo-inositol 3-kinase
				*Glyma.08g358700*	WD40 repeat-containing protein	Mannosyl-oligosaccharide alpha-1,3-glucosidase
				*Glyma.08g358800*	D-galactose detoxification	UDP-sugar pyrophosphorylase
				*Glyma.08g360400*	Sugar efflux transporter for intercellular exchange	12-oxophytodienoic acid reductase
				*Glyma.08g360500*	Sugar efflux transporter for intercellular exchange	12-oxophytodienoic acid reductase
				*Glyma.08g361200*	Sugar (and other) transporter	Sucrose transport protein SUC3 isoform
	ss715613179	12	5,486,355	*Glyma.12g072800*	Glycolysis I and II	-^f^
	ss715627820	17	41,098,767	*Glyma.17g257800*	Hexokinase	Glucose-6-phosphate isomerase
				*Glyma.17g258100*	Gibberellin regulated protein	Xyloglucan endotransglucosylase/hydrolase
	ss715627853	17	41,440,620	*Glyma.17g260300*	Peroxidase	Glycosyltransferases
				*Glyma.17g260400*	Rare lipoprotein A (RlpA)-like double-psi beta-barrel	Polygalacturonase precursor
	ss715636857	20	1,907,881	*Glyma.20g017400*	GDP-fucose protein O-fucosyltransferase	O-fucosyltransferase 23 isoform
				*Glyma.20g018000*	GDP-glucose biosynthesis	UTP–glucose-1-phosphate uridylyltransferase isoform X2
				*Glyma.20g018200*	Glycosyl hydrolases family 17	Glycosyltransferases
	ss715637428	20	34,286,637	*Glyma.20g099600*	Methionyl-tRNA synthetase	Leucine–tRNA ligase
Stachyose	ss715583079	2	44,214,908	*Glyma.02g255100*	Glycosyl transferase	Xyloglucan galactosyltransferase
				*Glyma.02g255400*	Hs1pro-1 N-terminus	O-fucosyltransferase 20
	ss715583119	2	44,448,179	*Glyma.02g258000*	Response to freezing	Glycosyltransferases
				*Glyma.02g258200*	Clathrin propeller repeat	Phosphatidylinositol-3,4,5-trisphosphate 3-phosphatase
	ss715592340	5	2,207,089	*Glyma.05g024800*	Galactosyl transferase	Xyloglucan galactosyltransferase
				*Glyma.05g025300*	Ribulose-phosphate 3 epimerase family	Probable ribose-5-phosphate isomerase
				*Glyma.05g025400*	Xyloglucan fucosyltransferase	Xyloglucan galactosyltransferase
	ss715592442	5	2,369,980	*Glyma.05g026800*	Xylogalacturonan β-1,3-xylosyltransferase	Glycosyltransferase family 64 protein c4
				*Glyma.05g027100*	alpha/beta-Hydrolases superfamily protein	Sucrose synthase
	ss715591198	5	35,773,064	*Glyma.05g166800*	Mitochondrial outer membrane protein 25	Fructose-1,6-bisphosphatase
	ss715601133	8	2,382,910	*Glyma.08g028600*	UDP-α-D-galacturonate biosynthesis II (from D-galacturonate)	UDPglucose–hexose-1-phosphate uridylyltransferase/UDP-sugar pyrophosphorylase 1
	ss715603880	9	3,771,212	*Glyma.09g044100*	Uncharacterized conserved protein	Condensin-2 complex subunit
	ss715639178	9	30,134,957	*Glyma.09g124300*	Photosynthesis	–
	ss715606330	10	3,541,231	*Glyma.10g040000*	Glutathione S-transferase	Alpha-1,3-glucosyltransferase
				*Glyma.10g040700*	Xyloglucan biosynthesis	Xyloglucan 6-xylosyltransferase 2
	ss715615716	13	18,379,941	*Glyma.13g077900*	Vacuolar H+-ATPase V0 sector	V-type h+-transporting ATPase subunit d
	ss715614101	13	24,090,619	*Glyma.13g128300*	Sugar Kinase	DNA repair protein xrcc4
				*Glyma.13g128400*	Sugar Kinase	DNA repair protein xrcc4
				*Glyma.13g132700*	D-myo-inositol (1,4,5)-trisphosphate degradation	Inositol polyphosphate 6-/3-/5-kinase
				*Glyma.13g133800*	UDP-glucuronate decarboxylase	UDP-sugar pyrophosphorylase 1
	ss715617675	14	13,187,218	*Glyma.14g113100*	Polysaccharide binding	–
	ss715622806	15	51,630,810	*Glyma.15g275300*	Triose-phosphate Transporter family	Glucose-1-phosphate adenylyltransferase
				*Glyma.15g275400*	Glycosyl hydrolases family 28	Pectinesterase
				*Glyma.15g276700*	Uncharacterized protein family	Glucan endo-1,3-beta-glucosidase a6

The most adjacent candidate gene was selected if there was no candidate gene associated with the carbohydrate metabolic pathway.

^a^Single-nucleotide polymorphism; ^b^Chromosome; ^c^Gene model: Glycine max cv. Williams 82 reference-genome gene models version 2.0; ^d^Phytozome database; ^e^STRING database; ^f^Not available.

Furthermore, GO annotation of the 17 relevant candidate genes for sucrose content confirmed six biological processes, including GO:0045490 (pectin catabolic process), GO:0006012 (galactose metabolic process), GO:0008643 (carbohydrate transport), GO:0006096 (glycolytic process), GO:0006004 (fucose metabolic process), and GO:0005975 (carbohydrate metabolic process) (**Supplementary Table 7**). Seven molecular functions were also confirmed, including GO:0030570 (pectate lyase activity), GO:0004335 (galactokinase activity), GO:0051119 (sugar transmembrane transporter activity), GO:0005366 (myo-inositol:proton symporter activity), GO:0004340 (glucokinase activity), GO:0005536 (glucose binding), and GO:0042973 (glucan endo-1,3-beta-D-glucosidase activity) (**Supplementary Table 7**).

Out of 155 genes, 24 relevant candidate genes for stachyose content were closely associated with carbohydrate metabolism (**Table 4**). Since a stachyose-associated significant SNP, ss715639178, showed no LD block range, the most adjacent gene, *Glyma.09g124300*, was selected as a candidate gene. Also, due to no candidate gene related to carbohydrate metabolism within the LD blocks of ss715603880 and ss715615716, the most adjacent genes, *Glyma.09g044100* and *Glyma.13g077900*, were selected, respectively. The largest number of relevant candidate genes (four) were found in the LD block of ss715614101 on Chr. 13, where carbohydrate metabolism-related genes, *Glyma.13g128300* (sugar kinase), *Glyma.13g128400* (sugar kinase), *Glyma.13g132700* (D-myo-inositol (1,4,5)-trisphosphate degradation), and *Glyma.13g133800* (UDP-glucuronate decarboxylase), are located. Also, nine genes, *Glyma.02g255100*, *Glyma.05g024800*, *Glyma.05g025300*, *Glyma.05g025400*, *Glyma.05g026800*, *Glyma.08g028600*, *Glyma.10g040700*, *Glyma.14g113100*, and *Glyma.15g275400*, are involved in carbohydrate metabolism, while eight genes, *Glyma.02g255400*, *Glyma.02g258000*, *Glyma.02g258200*, *Glyma.05g027100*, *Glyma.05g166800*, *Glyma.10g040000*, *Glyma.15g275300*, and *Glyma.15g276700*, have functional interactions with carbohydrate metabolism-related proteins.

Furthermore, GO annotation of the 24 relevant candidate genes for stachyose content confirmed seven biological processes, including GO:0006486 (protein glycosylation), GO:0009969 (xyloglucan biosynthetic process), GO:0019323 (pentose catabolic process), GO:0006012 (galactose metabolic process), GO:0019252 (starch biosynthetic process), GO:0042732 (D-xylose metabolic process), and GO:0005975 (carbohydrate metabolic process) (**Supplementary Table 7**). Seven molecular functions were also confirmed, including GO:0016757 (glycosyltransferase activity), GO:0008107 (galactoside 2-alpha-L-fucosyltransferase activity), GO:0004335 (galactokinase activity), GO:0019200 (carbohydrate kinase activity), GO:0048040 (UDP-glucuronate decarboxylase activity), GO:0030247 (polysaccharide binding), and GO:0004650 (polygalacturonase activity) (**Supplementary Table 7**).

### Tissue-specific gene expression analysis for relevant candidate genes

**Figure 5:**
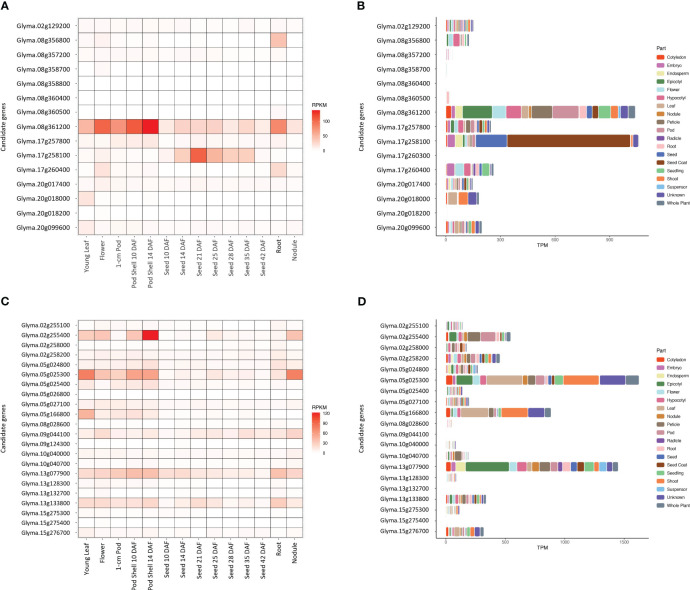
Gene expression levels of the potential candidate genes in 14 different tissues and 19 parts of soybean plant for sucrose content (**A**, **B**, respectively) and stachyose content (**C**, **D**, respectively). The raw gene expression counts were normalized using a Reads Per Kilobase of transcript per Million mapped reads (RPKM) and a Transcripts Per Million (TPM) methods.

Two genes, *Glyma.08g361200* and *Glyma.17g258100*, related to sucrose content had distinctively higher gene expression levels of 160 and 273 RPKM, respectively, than other candidate genes in the developing seeds from Seed 10 DAF to Seed 42 DAF (**Figure 5A**). Furthermore, these two genes showed the highest total TPM values (1,041 and 1,061 TPM, respectively) across 19 plant parts in soybean (**Figure 5B**). *Glyma.17g258100* especially had considerably higher gene expression levels in seed and seed coats, while the gene expression of *Glyma.08g361200* was distributed throughout the plant. The gene expression data of *Glyma.12g072800* and *Glyma.17g260300* (**Figure 5A**) and *Glyma.08g358800* and *Glyma.12g072800* (**Figure 5B**) was not available.

Four genes, *Glyma.05g025300*, *Glyma.09g044100*, *Glyma.13g077900*, and *Glyma.13g133800*, related to stachyose content, showed relatively higher gene expression levels (58, 76, 99, 71 RPKM, respectively) than other candidate genes in the developing seeds from Seed 10 DAF to Seed 42 DAF (**Figure 5C**). *Glyma.05g025300* had the highest total gene expression level (1,625 TPM), followed by *Glyma.13g077900* (1,450 TPM) across 19 plant parts in soybean (**Figure 5D**). *Glyma.05g025300* had higher gene expression levels in leaf and shoots, while *Glyma.13g077900* had a higher gene expression level in the epicotyl (embryonic shoot). The gene expression data of *Glyma.13g128400* and *Glyma.14g113100* (**Figure 5C**) and *Glyma.05g026800*, *Glyma.09g124300*, *Glyma.13g128400*, and *Glyma.14g113100* (**Figure 5D**) was not available.

## Discussion

In the current study, the sucrose and stachyose content showed significant phenotypic variations across the environments studied (**Table 1**). Similar to other seed composition traits, such as protein and oil, soluble carbohydrates are also influenced by diverse environmental factors, especially temperatures during pod-filling stages (Bandillo et al., 2015; Bilyeu and Wiebold, 2016). In a previous study, Bilyeu and Wiebold (2016) reported cooler temperatures during pod-filling stages increased sucrose content while decreasing stachyose content in soybeans. Among the environments studied, the POR_20 showed relatively cooler temperatures compared to the others during the pod-filling stages, while the STU_15 showed relatively warmer temperatures than the others (**Supplementary Figure 4**). The sucrose content of the POR_20 was the highest, and that of the STU_15 was the lowest, which followed the well-known relationship between temperature and sucrose content (Jo et al., 2018; Jo et al., 2019). On the other hand, the trend of stachyose content across environments was less influenced by temperature conditions, which the result was in good agreement with earlier reports by Kumar et al. (2010); Jo et al. (2018), who concluded that stachyose content was more genotype dependent. In future investigations, more geographically dispersed locations will be required to extensively test potential environmental influences on soluble carbohydrates.

Assessing multiple environment phenotypic datasets, a subset of 24 soybean accessions with either/both high sucrose and/or low stachyose content was selected for further genetic analysis and breeding applications (**Table 5**). Accessions PI 536547 B, PI 561288, and PI 549065 exhibited desirable soluble carbohydrate profiles with high sucrose (> 7.7%) and low stachyose content (< 2.3%), which are greatly beneficial to human and animal consumption (Jo et al., 2018; Sui et al., 2020). Field evaluations conducted in 2014 and 2015 showed these PI lines had larger seed sizes (> 29.0 g per 100 seeds) and high protein content (> 42.0%) on a dry weight basis. In particular, larger seed sizes and high sucrose and high protein content are essential quality parameters in soy-food production, including edamame, miso, and tofu (Konovsky et al., 1994; Zeipina et al., 2017; Jegadeesan and Yu, 2020). Interestingly, the soybean accessions with higher sucrose in this panel mainly originated from Japan. This indicated the historical selection in Japan was conducted based on the taste of soybean products for human uses, such as edamame, miso, natto, soy sauce, and tofu, of which sucrose is a main contributor to the sweetness of soybean products (Kaga et al., 2012; Rosset et al., 2012; Sui et al., 2020; Wang et al., 2023).

**Table 5:** The list of promising soybean germplasm resources to modify soluble carbohydrate profiles for human and animal uses.

Name	Maturity Group	Origin	Sucrose^a^	Stachyose^a^	Protein^b^	Seed size^b^
			Mean ± SD^c^ (%)			(g/100 seeds)
PI 506530	VI	Japan	8.8 ± 0.6	4.1 ± 0.5	40.6 ± 1.7	26.0 ± 3.9
PI 506903	IV	Japan	8.6 ± 0.4	3.9 ± 0.4	40.9 ± 1.9	32.6 ± 1.7
PI 506937	IV	Japan	8.2 ± 0.5	3.5 ± 0.7	36.7 ± 1.6	28.7 ± 2.5
PI 229343	IV	Japan	8.0 ± 0.6	4.0 ± 0.5	39.2 ± 1.7	31.5 ± 2.1
PI 507449	IV	Japan	7.9 ± 0.6	2.6 ± 0.8	46.0 ± 1.8	26.8 ± 2.2
PI 507438	VI	Japan	7.8 ± 0.7	3.8 ± 0.8	40.1 ± 2.1	28.7 ± 2.6
PI 398925	VI	South Korea	7.8 ± 0.7	5.1 ± 0.4	42.1 ± 1.8	23.1 ± 1.9
PI 536547 B	III	Taiwan	7.8 ± 0.5	1.9 ± 0.5	43.4 ± 1.4	29.7 ± 3.1
PI 506593	VI	Japan	7.7 ± 0.9	4.8 ± 0.5	41.1 ± 2.1	24.9 ± 3.5
PI 561288	IV	Taiwan	7.7 ± 0.8	2.3 ± 0.7	42.7 ± 1.0	30.3 ± 3.9
PI 548486	VI	Japan	7.7 ± 0.7	5.0 ± 0.6	42.2 ± 2.5	28.2 ± 2.5
PI 416892	III	Japan	7.7 ± 0.6	3.8 ± 0.6	44.9 ± 1.4	32.8 ± 2.9
PI 507523	III	Japan	7.7 ± 0.6	2.5 ± 0.5	46.3 ± 1.0	31.4 ± 2.1
PI 549065	IV	Japan	7.7 ± 0.5	2.1 ± 0.5	43.0 ± 1.2	33.7 ± 3.3
PI 424590 A	IV	South Korea	7.6 ± 0.5	3.8 ± 0.7	38.9 ± 1.2	27.4 ± 1.3
PI 561292 B	IV	Taiwan	7.2 ± 0.5	2.2 ± 1.0	42.7 ± 1.0	33.0 ± 3.2
PI 507273	III	Japan	6.9 ± 0.6	2.5 ± 0.7	40.7 ± 1.2	23.1 ± 1.6
PI 506801 B	III	Japan	6.8 ± 0.7	2.3 ± 0.7	43.2 ± 1.5	28.0 ± 0.9
PI 507487	III	Japan	6.8 ± 0.6	2.4 ± 0.6	43.4 ± 1.5	28.7 ± 2.1
PI 196162	III	Japan	6.8 ± 0.3	2.5 ± 0.5	42.7 ± 0.6	25.2 ± 1.2
PI 506800 A	III	Japan	6.7 ± 0.6	1.8 ± 0.4	45.3 ± 1.1	29.8 ± 0.8
PI 506800 B	III	Japan	6.6 ± 0.5	2.1 ± 0.6	43.8 ± 1.2	29.2 ± 1.4
PI 506799	III	Japan	6.4 ± 0.7	2.2 ± 0.7	44.1 ± 1.4	28.1 ± 1.2
PI 506801 A	III	Japan	5.5 ± 0.5	2.1 ± 0.5	45.4 ± 1.2	26.2 ± 0.9

^a^Traits evaluated across all locations (FAY_14, STU_14, FAY_15, STU_15, POR_20, COL_21, and POR_21); ^b^Traits evaluated only across limited locations (FAY_14, STU_14, FAY_15, and STU_15); ^c^Standard deviation.

The current bottleneck of narrowed genetic diversity has been addressed by soybean breeders for decades. Breeding and commercializing soybean cultivars with desirable soluble carbohydrate profiles has been slowed down due to the limitation of genetic sources, although the importance has been continuously expressed to meet global premium demand. Besides those PI lines mentioned above, we employed two additional accessions, PI 506937 and PI 506593, with desirable carbohydrate profiles for developing bi-parental mapping populations in an on-going effort to characterize the genetic architecture of carbohydrate composition traits. We anticipate these PI lines could be useful as novel genetic resources for breeders to develop new specialty soybean cultivars with modified soluble carbohydrate content.

For decades, different analysis models have been developed and utilized for GWAS. Among these, MLM is one of the most popular models, which incorporates population structure and kinship as covariates to reduce false positives and increase the statistical power in identifying significant marker-trait associations (Yu et al., 2005; Ficht et al., 2022). In the current study, the MG effect was significant on sucrose and stachyose content (**Supplementary Table 3**). Therefore, the corresponding MG of each genotype was additionally incorporated as a categorical covariate to reduce the potential bias derived from the MG differences (**Supplementary Table 1**). The relatively smaller panel size (< 300) could be a limitation for GWAS, although it contains four MGs and six origins. To declare a significant SNP, we tested a typical Bonferroni Correction using a stringent threshold (-log_10_(P) = 5.6); however, no significant SNP was detected. Thus, a general consensus value of 0.001 (-log_10_(P) = 3.0) was used as a significant cut-off value to detect significant SNPs (Hwang et al., 2014).

Using phenotypic data of each environment and estimated BLUP, seven significant SNPs for sucrose content were identified across five chromosomes (Chrs. 2, 8, 12, 17, and 20) (**Figure 2** and **Table 2**). Among them, four SNPs were confirmed to be close to previously reported sucrose-related genomic regions. Hu et al. (2023) reported a significant SNP (-log_10_(P) = 3.3) associated with fructose, which was located in the same region as ss715581183 at 13,523,639 bp on Chr. 2. Fructose is one of the monosaccharides that comprise the sucrose molecule. Lu et al. (2022) reported a significant SNP (-log_10_(P) = 8.8) associated with total soluble carbohydrate at 5,036,567 bp on Chr. 12, which was mapped approximately 449 Kbp downstream of ss715613179 (5,486,355 bp). Total soluble carbohydrates were positively correlated with sucrose in soybeans (Hou et al., 2009). Also, Kim et al. (2006) reported a sucrose-related QTL (‘Seed Sucrose 3-4’ at Soybase, LOD = 8.4) associated with the marker Satt442 at 6,390,806 - 6,391,062 bp on Chr. 12, which is mapped approximately 904 Kbp upstream of ss715613179. Based on only 110 markers used by Kim et al. (2006), the distance of less than a million bp between the two markers was considered significant. Patil et al. (2018) reported a sucrose-related QTL, qSUC_20 (LOD = 3.8), at 2,386,021 - 2,558,940 bp on Chr. 20, which was mapped approximately 478 Kbp upstream of ss715636857 (1,907,881 bp). Wang et al. (2023) also reported a sucrose-related SNP (-log_10_(P) = 2.9) at 34,981,501 bp on Chr. 20, which was mapped approximately 690 Kbp upstream of ss715637428 (34,286,637 bp). Another marker, Satt270 (35,362,576 - 35,362,794 bp), on Chr. 20 was reported as an associated marker to a sucrose-related QTL (LOD = 1.9). It was mapped approximately 1 Mbp upstream of ss715637428 (Wang et al., 2014). Three SNPs, ss715602502 on Chr. 8, ss715627820 and ss715627853 on Chr. 17, are novel for sucrose content in soybean.

Thirteen significant SNPs for stachyose content were identified across eight chromosomes (Chrs. 2, 5, 8, 9, 10, 13, 14, and 15) (**Figure 3**; **Table 2**). Unlike sucrose, only one SNP was confirmed to be close to the previously reported stachyose/oligosaccharides-related genomic regions. Kim et al. (2005) reported a stachyose-related QTL marker (‘Seed Oligosaccharide 1-1’ at Soybase, LOD = 7.7) associated with the marker Satt546 at 40,699,300 - 40,699,539 bp on Chr. 2, which was mapped approximately 3.5 Mbp downstream of ss715583079 (44,214,908 bp). The other 12 SNPs identified in this study have not been previously reported and were considered novel SNPs for stachyose content in soybean. Historically, soybean breeding programs have been more interested in sucrose than stachyose, resulting in more than twice as many sucrose-related QTL/SNPs published in the Soybase (https://soybase.org).

In the current study, ss715602502 on Chr. 8 was the most consistent SNP significantly associated with sucrose content, followed by ss715613179 on Chr. 12 and ss715627853 on Chr. 17 (**Figure 2**; **Supplementary Table 4**). However, ss715602502 had no significant allelic effect on sucrose content (**Figure 4A**). This phenomenon has been explained by the fact that not all variants are causally associated with a trait, and the association can be indirect (MacArthur et al., 2014; Gallagher and Chen-Plotkin, 2018; Schaid et al., 2019). Despite no significant allelic effect of ss715602502, the estimated LD block contained seven candidate genes underlying carbohydrate transport/metabolism (**Table 4** and **Supplementary Tables 6, 7**). Among these, two genes, *Glyma.08g360400* and *Glyma.08g360500*, were reported to belong to the SWEET (Sugars Will Eventually be Exported Transporters) gene family (SWEET25 and SWEET26, respectively), which play essential roles in transporting glucose molecules across a membrane (Patil et al., 2015). The most consistent significant SNP for stachyose content, ss715592442 on Chr. 5, was identified in seven environments. The SNP ss715583119 on Chr. 2 was identified in six environments (**Figure 3**; **Supplementary Table 4**). Like sucrose, ss715592442 and ss715583119 had no significant allelic effect on stachyose content. The estimated LD blocks contained carbohydrate metabolism-related genes, including glycosyltransferase family 64 protein c4 (*Glyma.05g026800*), sucrose synthase (*Glyma.05g027100*), glycosyltransferases (*Glyma.02g258000*), and clathrin propeller repeat (*Glyma.02g258200*) (**Figure 4B**; **Table 4**). The biological process of *Glyma.02g258200* is to route acidic α-galactosidases to protein storage vacuoles, and it is known to facilitate the accumulation of RFOs during seed development in pea (Blöchl et al., 2008). Therefore, integrative post-GWAS analyses, including protein interaction, metabolic pathway, biological function, cellular component, and molecular function, are crucial to comprehensively understand the variants/candidate genes identified in GWAS (Jia et al., 2011).

Two candidate genes associated with sucrose, *Glyma.08g361200* and *Glyma.17g258100*, showed high gene expression levels in the developing seed tissues and total expression levels across 19 different parts of the soybean plant (**Figures 5A, B**). The molecular function of *Glyma.08g361200* is myo-inositol:proton symporter activity, which is responsible for transferring myo-inositol from one side of a membrane to the other (**Supplementary Table 7**). Myo-inositol is essential for galactinol biosynthesis, and galactinol plays a key role in the chain elongation of sucrose to RFOs (Saravitz et al., 1987; Irvine and Schell, 2001). However, no information was available for *Glyma.17g258100* in the GO database. Instead, based on Phytozome and STRING database, it is responsible for gibberellin-regulated protein and protein interaction with xyloglucan endotransglucosylase/hydrolase (**Table 4**). Xyloglucan endotransglucosylase/hydrolase catalyzes the cleavage of the main chain of xyloglucan molecules until only glucose, xylose, and galactose are produced, of which glucose is an essential component of the sucrose metabolism (Buckeridge, 2023).

Two candidate genes, *Glyma.05g025300* and *Glyma.13g077900*, showed high total gene expression levels for stachyose content in developing seed tissues and total expression levels across 19 different parts of the soybean plant (**Figures 5C, D**). Unlike sucrose, these genes showed relatively higher gene expression levels in leaf, shoot, and epicotyl (embryonic shoot) than in seed and seed coats. This suggested that stachyose content may be affected by genes expressed not only in the developing seed but also in aerial parts, including leaf and shoot. The raffinose synthase 3 gene (*Glyma.05g003900*, Wm82.a2.v1), widely used in soybean breeding programs to reduce RFOs, also has the highest expression level in shoots, followed by petioles and leaves in soybean (**Supplementary Figure 5**). The gene, *Glyma.05g025300*, is responsible for the carbohydrate metabolic process (GO:0005975), while *Glyma.13g077900* is responsible for the proton-transporting V-type ATPase (GO:0033179) (**Supplementary Table 7**). In plants, V-type ATPase plays an important role in regulating signaling events in response to diverse environmental stimuli (Elmore and Coaker, 2011). Therefore, this confirmed that stachyose is also known to play an important role in protection against abiotic stress, such as salinity, cold, and drought in plants (Mundree et al., 2002; ElSayed et al., 2014; Zhang et al., 2019; Yan et al., 2022).

To date, three major genes, *Glyma.05g003900* (raffinose synthase 3, RS3), *Glyma.06g179200* (raffinose synthase 2, RS2), and *Glyma.11g238800* (D-myo-inositol-3-phosphate synthase 1, MIPS1), primarily responsible for increased sucrose and reduced RFOs, have been identified. Even though molecular markers related to these genes have been utilized for marker-assisted selection (MAS) breeding, a limited number of soybean cultivars for human and animal consumption have been developed and commercialized. Recently, Benson Hill, Inc. (https://bensonhill.com/) announced the release of a new soybean cultivar with ultra-high protein and low RFOs content, coupled with other improved agronomic traits. In this study, we reported new genomic regions harboring candidate genes associated with increased sucrose and reduced stachyose, which did not overlap with the known genes, RS2, RS3, and MIPS1. Therefore, the promising soybean germplasms and novel genetic sources newly identified in this study would be critical to broadening germplasm resources and enriching our understanding of the genetic architecture for the development of new soybean cultivars for animal feed efficiency and natural sweetness in human consumption. Future studies, including genome-editing technologies, a fine-mapping strategy, and molecular marker development, will be required for the functional validation of the novel findings in this study.

## Conclusion

This study identified three and 12 novel SNPs associated with sucrose and stachyose content, respectively, through a GWAS using 220 soybean accessions. Four novel candidate genes for sucrose (*Glyma.08g361200* and *Glyma.17g258100*) and stachyose (*Glyma.05g025300* and *Glyma.13g077900*) content were further identified. We also reported three promising lines (PI 536547 B, PI 561288, and PI 549065) as germplasm resources that can be valuable for developing new soybean cultivars with desirable soluble carbohydrate profiles. The novel discoveries in this study will extend the current knowledge of the genetic basis underlying sucrose and stachyose content in soybean seed. Overall, new genetic resources will benefit soybean breeders in modifying carbohydrate profiles to meet the high demand for animal and human consumption.

## Data availability statement

The datasets presented in this study can be found in online repositories. The names of the repository/repositories and accession number(s) can be found in the article/**Supplementary Material**.

## Author contributions

DL: Data curation, Formal Analysis, Investigation, Methodology, Writing – original draft, Writing – review & editing. LL: Data curation, Formal Analysis, Investigation, Methodology, Writing – review & editing. DM: Formal Analysis, Methodology, Data curation, Investigation, Writing – review & editing. TV: Conceptualization, Data curation, Formal Analysis, Investigation, Methodology, Supervision, Writing – original draft, Writing – review & editing. GS: Conceptualization, Funding acquisition, Methodology, Project administration, Supervision, Writing – original draft, Writing – review & editing. DX: Methodology, Writing – review & editing. HN: Conceptualization, Funding acquisition, Methodology, Project administration, Supervision, Writing – original draft, Writing – review & editing.
