# Supplementary figures and images for: Novel genetic resources associated with sucrose and stachyose content through genome-wide association study in soybean (Glycine max (L.) Merr.)
Source: Front Plant Sci. 2023 Nov 1;14:1294659. doi: 10.3389/fpls.2023.1294659 (PMC10646508; doi:10.3389/fpls.2023.1294659)

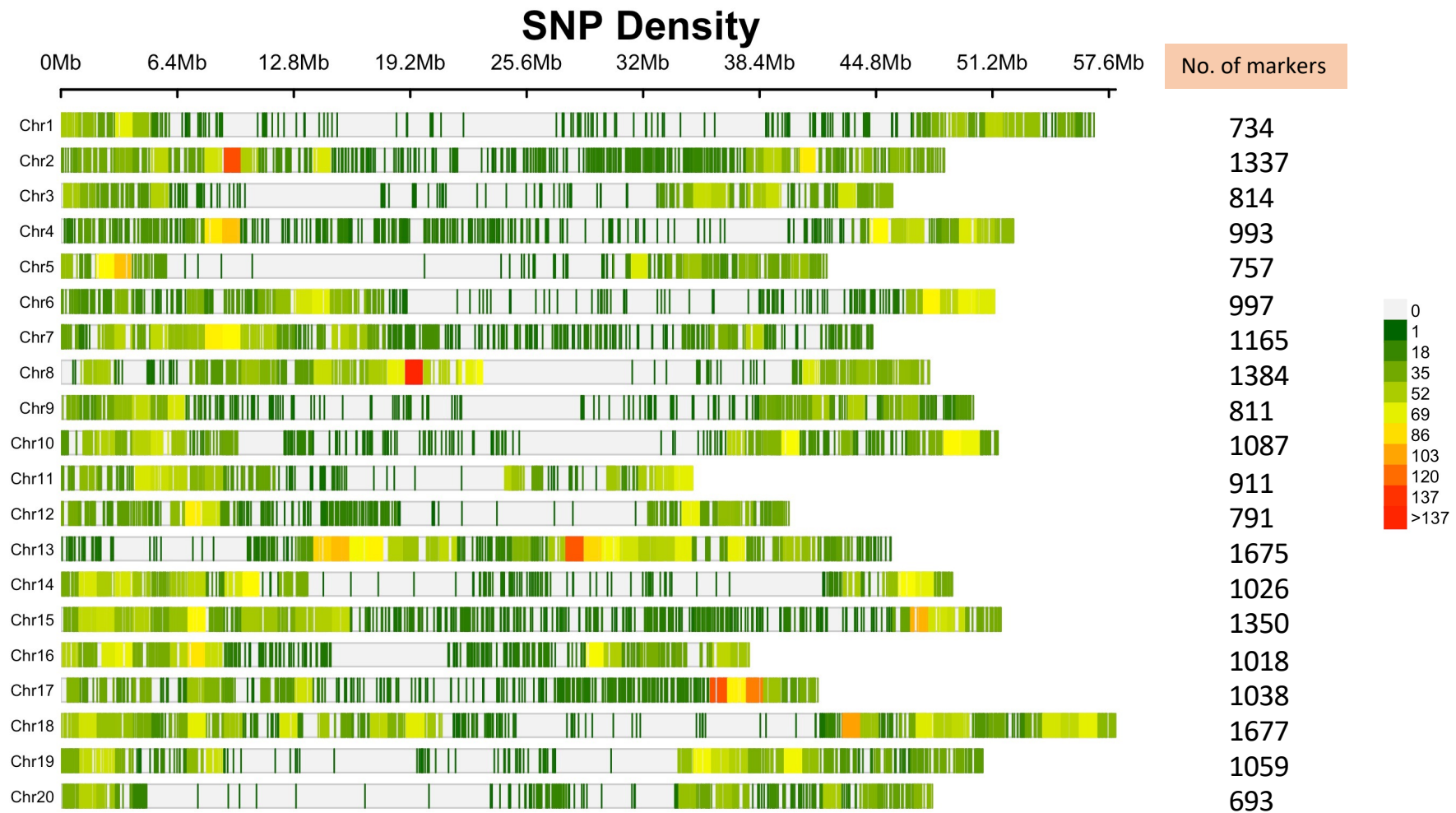

Supplementary Figure 1. The number of filtered SNPs mapped across 20 soybean chromosomes

Supplement: Supplementary file 1 [file DataSheet_1.pdf]
