# Supplementary material for: Novel genetic resources associated with sucrose and stachyose content through genome-wide association study in soybean (Glycine max (L.) Merr.)
Source: Front Plant Sci. 2023 Nov 1;14:1294659. doi: 10.3389/fpls.2023.1294659 (PMC10646508; doi:10.3389/fpls.2023.1294659)
Supplement: Supplementary file 4 [file DataSheet_4.pdf]

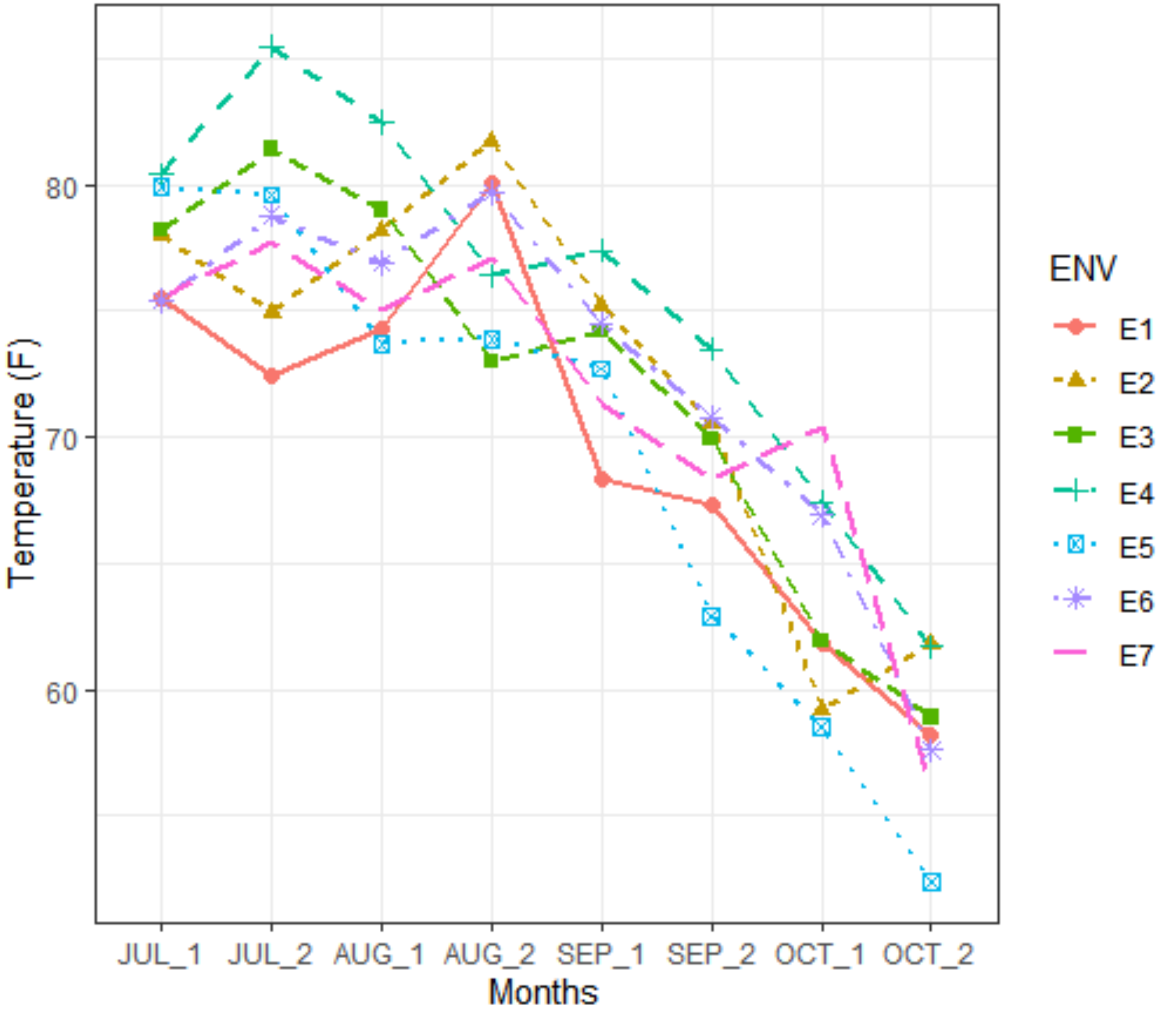


Supplementary Figure 4. Temperatures during soybean pod-filling stages (July - October) across environments. '_1' and '_2' indicate the first 15 days and the rest of the month, respectively. E1, Fayetteville in 2014; E2, Stuttgart in 2014; E3, Fayetteville in 2015; E4, Stuttgart in 2015; E5, Portageville in 2020; E6, Columbia in 2021; E7, Portageville in 2021.
