# Supplementary material for: Novel genetic resources associated with sucrose and stachyose content through genome-wide association study in soybean (Glycine max (L.) Merr.)
Source: Front Plant Sci. 2023 Nov 1;14:1294659. doi: 10.3389/fpls.2023.1294659 (PMC10646508; doi:10.3389/fpls.2023.1294659)
Supplement: Supplementary file 5 [file DataSheet_5.pdf]

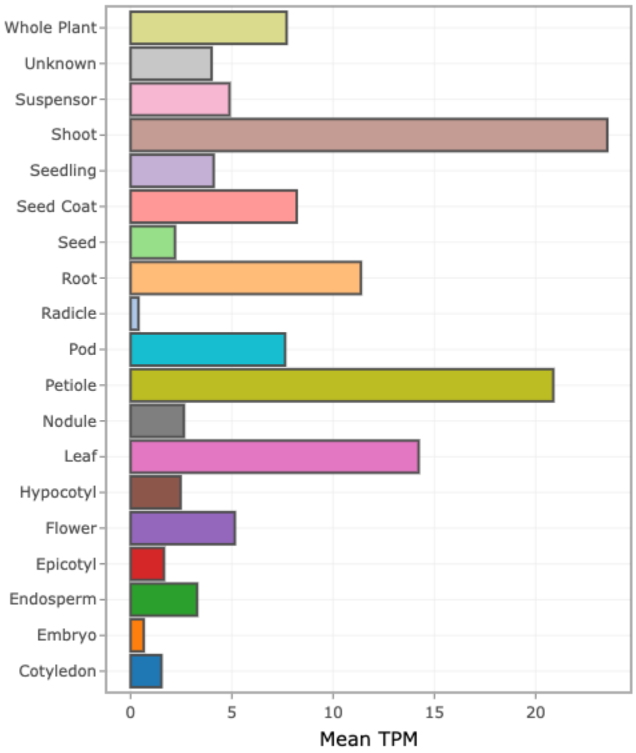

Supplementary Figure 5. The gene expression level of raffinose synthase 3 gene (*Glyma.05g003900*, Wm82.a2.v1) across 19 different parts of the soybean plant. The raw gene expression counts were normalized using a Transcripts Per Million (TPM) methods.
